# PETG as an Alternative Material for the Production of Drone Spare Parts

**DOI:** 10.3390/polym16212976

**Published:** 2024-10-24

**Authors:** Marija Z. Baltić, Miloš R. Vasić, Miloš D. Vorkapić, Danica M. Bajić, Ján Piteľ, Petr Svoboda, Aleksandar Vencl

**Affiliations:** 1 University of Belgrade, Faculty of Mechanical Engineering, Kraljice Marije 16, 11120 Belgrade, Serbia; mbaltic@mas.bg.ac.rs; 2 Institute for Testing of Materials, Bulevar Vojvode Mišića 43, 11000 Belgrade, Serbia; milos.vasic@institutims.rs; 3 University of Belgrade, Institute of Chemistry, Technology and Metallurgy, National Institute of the Republic of Serbia, Njegoševa 12, 11000 Belgrade, Serbia; worcky@nanosys.ihtm.bg.ac.rs; 4 Military Technical Institute, 11030 Belgrade, Serbia; danica.bajic@mod.gov.rs; 5 Faculty of Manufacturing Technologies, Technical University of Košice, Bayerova 1, 080 01 Prešov, Slovakia; jan.pitel@tuke.sk; 6 Faculty of Mechanical Engineering, Brno University of Technology, Technická 2896/2, 616 69 Brno, Czech Republic; petr.svoboda@vut.cz

**Keywords:** additive manufacturing, PETG, drone, microhardness, abrasive wear, toughness, tensile strength, impact test

## Abstract

Material selection is the main challenge in the drone industry. In this study, hardness, abrasive wear, impact resistance, tensile strength, and durability (frost resistance and accelerated ageing) were identified as important characteristics of drone materials. The additive manufacturing technology was used to produce the drone leg specimens and prototype. The suitability of PETG as a primary filament material in the design of the drone leg was investigated. Nine series were printed with different raster lines (0.1, 0.2 and 0.3 mm) and infill densities (30, 60 and 90%). Printed specimens were annealed in salt and alabaster, as well as immersed in liquid nitrogen. Series with raster line-infill densities of 0.1–30, 0.3–30, 0.1–90 and 0.3–90 were identified as the most interesting ones. Thermally treated specimens had better mechanical and durability properties, and infill density was found to be the most important printing parameter. Specimen annealed in salt with a raster line of 0.1 mm and infill density of 90% had the best results. Since ABS is the most common material used for drone leg production, its properties were compared with the PETG specimen, which showed the best properties. The potential of PETG as an alternative material was proven, while the flexibility, productivity and suitability of the leg drone design were additionally confirmed.

## 1. Introduction

The exponential growth of drones was reported worldwide during the last decade. The first design request for drone parts is to define a set of necessary characteristics, including the usage of recycled materials. The absence of international standards which specify the characteristics and performance requirements for drone parts is seen as an acute problem. Material selection is still one of the main challenges in the drone industry. The main parts of every drone are the frame, propellers, motors, electronics, battery, camera and sensors [1]. Each of these parts uses a specific group of adopted or defined materials. Aluminium, carbon fibre and thermoplastic filaments are the most widely used materials. The frame is mostly made of carbon fibre, aluminium and plastic. Propellers are manufactured of carbon fibre and plastic, while engines contain copper, aluminium, brass, bronze and steel. Aluminium is a lightweight material resistant to corrosion and it has high strength capability. It is used for more expensive and heavier drone models. Thermoplastic materials are inexpensive and light, but less durable and rigid compared to others. Factors such as drone weight, strength, and load capacity are decisive parameters for the consideration of drone leg material. The weight of the material is an essential feature because a lighter material will lower the total weight of the drone, which significantly affects the flight duration and enables quick drone manoeuvre. Also, a lighter drone allows for quicker lift-off and longer battery life [2]. Carbon fibre offers a high stiffness-to-weight ratio, ensuring the stability and controllability of the drone during flight [3].

Even though fused deposition modelling (FDM) is a relatively new technology, it was well accepted in the aerospace industry, healthcare, dentistry, medicine, engineering, civil construction, education, arts, etc., since prototypes or small parts with complex geometry and high precision can be easily produced [3,4,5,6]. Cost, mechanical properties, hardness, wear resistance and durability are the most important characteristics of materials, while for eco-friendliness design flexibility, productivity and repeatability are crucial parameters for the selection of thermoplastic filaments [6,7]. Maximum take-off weight, speed and altitude were identified in EU regulation 2019/947 as limiting flying requirements for drone application. This regulation has accurately and decisively defined the specifications for drone classification based on the drone weight. Drone testing is still an unregulated and unharmonized area of EU legislation. There are some common manufacturers’ requests for drone testing based on its intended use in moderate, severe and extreme environmental conditions. In addition to crash tests, impact tests, vibration tests and various durability tests (IC, UV, extreme temperature, chemical resistance, humidity, etc.), navigation and signal data processing tests are used in the drone industry for device specification. The performance requirement of drone parts is in direct correlation with the properties of the used material. Until now, the necessary properties of drone materials were not listed or uniformed in any legislative document. This situation promotes the unharmonized approach and leads to the inability to precisely rank various materials for the manufacturing of drone parts.

The technical characteristics of the printer device (maximal operating temperature and printing speed) and filament properties (temperature resistance and viscosity) are decisive factors for printing failure reduction since their level is responsible for the continuous flow of the molten material throughout the printer nozzle. If the temperature is not properly set and its values are higher than required, decomposition of the polymer is expected. This correspondingly leads to a decrease in the quality of the printed parts [4,8].

The commonly used thermoplastic filament raw materials with exceptional flexibility, abrasion resistance and elasticity are acrylonitrile butadiene styrene (ABS), polylactic acid (PLA) and polyethylene terephthalate glycol (PETG). Until now, ABS was preferred as the basic material for drone parts [9], while the PLA was mostly used in drone-supporting structure realisation [10,11]. Even though ABS, PLA and PETG belong to the same “workability” group, their modulus of elasticity and maximal stress significantly differs. If the load is applied in the direction of the printing layers, the PETG specimens have higher strength than the ABS specimens, while the opposite situation is registered for the compression test [10]. Unlike ABS, PETG demonstrates superior adhesion with the print surface, eliminating the need for heating the bed chamber at 110 °C. The plate temperatures for PETG printing are in the range of 60 to 80 °C [12]. The manufacturing process is more efficient and reliable since PETG-printed parts have much fewer temperature-related print defects than ABS. This means that the risk of warping, deforming, peeling, and printed parts lamination is significantly reduced [13]. The UV resistance of PETG specimens is much higher, whereas the opposite situation is registered for temperature resistance [14]. In addition, during PETG printing, less odour and lower volatile compounds and particle emissions are released, labelling it as a more suitable and environmentally friendly material. PETG is quite affordable and is generally accessible as a spool filament form in various colours. It is also a very stable, durable and resistant material [15,16]. This was also confirmed by Tunalioglu and Agca [17], who compared the printed gear wheels produced from commercial ABS, PLA and PETG. It was also shown that ABS and PLA have similar wear behaviour.

The temperature-strengthening effect on the mechanical properties of PETG and PETG composites was reported by Valvez et al. [18]. They concluded that higher temperatures and longer exposure time resulted in higher hardness, which is approximately 20% higher in the case of treated material. In addition, the bending strength and modulus were increased by 11 and 17%, respectively, in comparison to the untreated material. Many authors investigated the effect of printing parameters like infill pattern, density and material type on the properties of printed structures [19,20]. Annealing temperatures, exposure times and printing parameters (speed, raster line, raster angle, infill density and extrusion temperature) were investigated on PLA specimens by Hasan et al. [21]. The most important conclusion was that higher temperatures and longer exposure times have a larger influence on mechanical properties. The increase was around 21% compared to the untreated material. In addition, the influence of the printing orientation on the plasticity of the specimens has shown that specimens with a raster angle of 0/90° are the most brittle, while the ones with a raster angle of −45/45° withstand the highest deformations before break [21]. Grasso et al. [22] have found that the mechanical properties of PLA specimens are directly correlated with raster angle and applied temperature. Durgashzam et al. [23] have investigated the influence of printing parameters (raster line, infill density and printing speed) on the flexural and tensile properties of PETG. Two regression equations were defined. The first was for the prediction of flexural strength while the second one was for the prediction of tensile strength. The raster line was found as the most important parameter which influenced the mechanical properties of PETG. The hardness properties of printed ABS, PLA and PETG without thermal strengthening are well researched. The measured hardness for PETG was in the range of 9.2 to 10 HV 50 [24]. Loskot et al. [25] have found that if the printing speed is larger than 60 mm/s, the generation of defects within and on the surface of printed specimens increases. The increase in hardness of PETG specimens in comparison to untreated material was around 20%. In the case of PLA annealed in alabaster, the increase in hardness was 12.7% [26].

Kumar et al. [27] investigated and analysed the impact of annealing on PETG and CFPETG (carbon fibre PETG) composites with different infill densities (25, 50, 75 and 100%). They concluded that annealed PETG specimens with 100% infill density had the highest mechanical properties. Annealed PETG and CFPETG at 100% infill density showed a 6–8% and 10–11% increase in mechanical properties compared to as-printed PETG and CFPETG, respectively. Srinidhi et al. [28] investigated the mechanical properties of annealed PETG and CFPETG specimens produced by FDM with different infill patterns (grid, honeycomb, rectilinear and cubic). The annealing temperature and expose time were 100 °C and 60 min, respectively. Authors found that PETG and CFPETG annealed specimens with the grid infill pattern had the highest stiffness, tensile strength, impact strength and flexural strength, with increases of 29, 27, 18 and 9%, respectively. Rangisetty and Peel [29] investigated PLA, ABS and PETG composites reinforced with carbon fibre and printed in three different infill patterns. The authors utilised three different annealing temperatures (65, 110 and 85 °C). The annealing process of 60 min has led to average increases in tensile strength of 29, 21, 15, 13 and 6% for PLA, PLACF, PETG, PETGCF, ABS and ABSCF, respectively. A significant increase in modulus of elasticity and flexural strengths was also confirmed for PETG and PETGCF.

The most important degradation agents are static heating, sub-zero exposure, thermal cycling, humidity exposure, water immersion, freeze–thaw and dry/wet cyclic conditions, the act of salt water, weathering, combined load and environmental exposure and chemical and UV radiation [30]. The listed agents act separately or simultaneously with the polymer matrix, and irreversible structural change occurs. Even though a good scientific background about the mechanisms involved in polymer degradation exists, a lack of procedures and standards for its determination is still evident. Most standards for measuring polymer degradation are focused only on a single degradation agent or one mechanism, which can sometimes lead to the wrong conclusions. The problem with assessing factors, for long-term polymer performance assessment, lies in the fact that their validity is questionable as they can sometimes be apparent [30]. Sedlak et al. [14] focused on determining the influence of degradation factors (humidity, temperature and UV) on the mechanical properties of PLA, PETG, ABS and ASA materials. After humidity degradation, a tensile stress decrease was found only for PLA and ABS materials. Schippers et al. [31] tested the mechanical properties of PLA using the combined effect of increased humidity and temperature. The decrease in the modulus of elasticity and ultimate strength was the highest when the humidity was at its maximal value. The opposite trend was registered for ductility. This was also confirmed by Aldeen and Owaid [32]. Bazli et al. [33] have investigated the action of freeze–thaw on glass fibre-reinforced polymers (GFRP). The 7% decrease in mechanical properties was found when specimens’ thawing was conducted in air. If water was used as a thawing agent, the decrease in mechanical properties was three times bigger due to the freezing and expansion of the absorbed water [33]. A similar observation was reported by Wu et al. [34].

The aim of this study was to explore the suitability of PETG as an ABS replacement material for the production of the drone spare part. Two thermal treatments (annealing in salt and annealing in alabaster) were applied and compared with as-printed conditions to improve the characteristics important for drone applications, such as hardness, abrasive wear resistance, impact resistance and tensile properties. Since drones can be used not only in the atmosphere but in the stratosphere or in the Arctic where temperatures are extremely low, the printed parts were also treated under extreme temperature conditions, i.e., a liquid nitrogen immersion treatment.

## 2. Materials and Methods

FDM, as a widely accepted additive manufacturing (AM) process, is used for the printing of ABS and PETG filaments. The corresponding manufacturer’s Devil Design (Mikolow, Poland) data sheets are reported elsewhere [35,36]. The FDM printer Original Prusa i3 MK3S+ (Prague, Czech Republic), whose characteristics are also specified elsewhere [37], with a working surface of 250 × 210 × 210 mm was used for specimen realisation. The printing process starts from a virtual model in which the geometry of the specimen is defined. The specified drawing file is converted into the G-code instruction set file, after which uploading enables the printing. The “open source” program Prusa Slicer V2.6.0 (Prague, Czech Republic) was used to generate the G-code. More detail can be found elsewhere [38,39]. In our case, the shape is precisely defined in the relevant testing standards. The filament is brought to the heater through the guide and gear system (the cold part of the extruder). In the heater, the filament is melted at a specific temperature and then it passes through the nozzle as a molten material. The molten filament is then deposited layer-by-layer on the printer plate and cooled to room temperature [40,41]. The nozzle with the same cylindrical cross-section shape was used in each experiment. Since the raster line was printed with the same device, using the same extruder, the strand effect was neglected.

For each printing combination, four ABS and thirty-six PETG cylinders were printed for hardness and wear testing. The diameter and height of the specimens were defined according to the abrasive wear standard [42] as 16 × 13 mm. For each printing combination, four ABS and sixteen PETG specimens were printed for tensile strength. The dog bone-shaped geometry with a total length of 150 mm, width of 20 mm, and thickness of 3 mm was used [43]. The number of specimens for the Charpy impact tests was the same as in the case of the tensile strength for both filaments. The rectangular specimens were cut from printed plates 100 × 100 mm. The length, width and thickness of the specimens were 10 × 60 × 5 mm, as specified in the standard [44]. The printing parameters are reported in Table 1.

Annealing in salt and alabaster was chosen since these treatments showed a beneficial effect on some PLA specimens [26], i.e., the investigated specimens had better structural properties and better mechanical properties after these treatments than the referent specimens. Thermal strengthening (annealing in salt and alabaster), for each printing combination, was applied only on 18 cylinders and 8 dog bone PETG specimens. The NaCl was grounded to the granulation of 63 µm, and the powder was dried at 60 °C for 18 h. After that, the specimens were placed in a salt mould. The annealing temperature was 230 °C for 30 min. The alabaster was first mixed with water. After that, the printed specimens were immersed in the alabaster mould and placed in an ultrasonic cleaner for 15 min. The mould was dried naturally for 24 h. The following step included oven drying for 3 h at 50 °C and additional annealing for 3 h at 200 °C. When the thermal process was over, all specimens were cooled at the rate of 7.5 °C/h. The Web Metallverarbeitung ball mill, Retsch sieve mesh 0.063 mm, analytical sieve Shaker AS200, Branson and Smith Kline, model B-220 and laboratory oven Carbolite Gero, model RHF 1500 were used. To test the material for extreme temperature, 9 cylinders and 4 dog bone PETG specimens were put in liquid nitrogen for 5 min. The MVE Cryo-Shipper canister was used to store 2 L of liquid nitrogen.

All identified necessary characteristics were experimentally determined on as-printed, annealed in salt and alabaster, and liquid nitrogen-immersed specimens. Since one of the objectives of this study was to scientifically approve or reject the use of PETG as an alternative to ABS, the first step was to find the limiting level of the necessary characteristics that the alternative material should have. The characterisation of as-printed ABS samples was the basic input for such consideration.

The microstructures of as-printed, annealed and liquid-immersed cylindrical specimens were first determined using an optical digital microscope Dino Lite Pro (AnMo Electronics Corporation, Taipei, Taiwan). Even though the salt and alabaster depositions were registered on the optical images, the used enlargements were not enough for the scientific confirmation of the salt and/or alabaster diffusion penetration pattern. The dual beam scanning electron microscope (SEM) with energy dispersive X-ray spectroscope (EDS) at 10 kV of acceleration voltage, type FESEM, Thermo Fisher Scientific FEI Scios 2 (Waltman, MA, USA) was primarily used to reveal the stated assumption. In addition, these techniques were also applied to analyse the dog bone specimen fracture after tensile testing. Since polymers are natural insulators, the preparation step for SEM/EDS microscopy requires the deposition of the thin conducting Au layer.

The characterisation of the as-printed and annealed specimens was evaluated also using differential scanning calorimetry (DSC) and Fourier-transform infrared spectroscopy (FTIR). DSC was recorded on TA Instruments Q20 (New Castle, DE, USA). Approximately 5 mg was placed in hermetically sealed aluminium pans. The specimens were heated from 20 to 180 °C at a rate of 10 °C/min, cooled to −50 °C at the rate of 10 °C/min, and then subjected to a second heating to 250 °C at the rate of 10 °C/min. The nitrogen flow rate was 50 mL/min. Indium was used as the reference for calibration of the equipment’s temperature.

FTIR mobile Alpha Bruker Optics device in attenuated total reflection mode, with a diamond as the crystal was used. The obtained spectra were analysed with the integrated OPUS software V8.2 developed by Bruker (Ettlingen, Germany).

The ASTM E384 [45] was used as a relevant test for hardness detection. HV = 1.845 × *P*/*D*^2^, where *P* is the used load and *D* is the average indentation length. The load of 0.245 N (25 gf) and hold time of 15 s were applied on the Vickers microhardness tester Time TH710 (Beijing, China). Wear resistance was determined according to ASTM G132 [42]. Specimen slides were fixed on abrasive paper, which was attached to the rotating roller and produces a helix sliding path. The normal load was 2.5 N, the sliding speed was 0.32 m/s and the total sliding distance was 40 m.

ISO 527-1 [46] was applied for the tensile test on all printed bone dog specimens. The test was carried out on the Instron 1122 universal test device with a TRC Pro acquisition system, with a maximum load capacity up to 5000 N. The dimensions of the specimens were as follows: the length between the clamps was 105 mm, thickness 31 mm, and width 102 mm. The test speed was set to 50 mm/min. The absorbed impact energy and toughness were determined on a Charpy impact pendulum Zwick (Karl Frank, GmbH, Leipzig, Germany) with a maximal impact energy of 7.5 J according to the EN ISO 179-2 standard [47].

**Figure 1:**
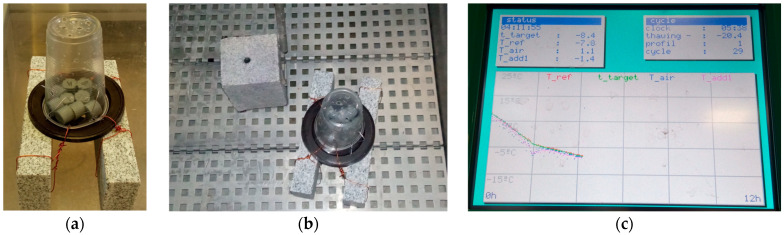
Frost resistance test: (**a**) preparation step, (**b**) climatic chamber, and (**c**) cycle monitoring.

Additional cylindrical specimens were printed for long-term durability freeze–thaw resistance tests. A slab test was used for freeze–thaw resistance determination, and the temperature was monitored and recorded continuously. The 3D-printed drone spare parts have to be durable in the temperature range from −20 to 50 °C. The absence of an international standard for determination of the freeze–thaw resistance of plastic materials has led to the use of EN 12371 [48]. Testing specimens were first dried at 30 °C until a constant mass was achieved. To prevent the uncontrolled movement of the specimens when the water level rose from the bottom of the testing device during the flooding cycle period, all specimens were placed on a plastic holder which was covered with a plastic cup. The copper wire was used to connect the previously drilled cup and holder with two stone supports (Figure 1a), and the stone block was placed in the centre of the testing device (Figure 1b). The main temperature probe was leaned on the block, while the auxiliary one was put into the centrally drilled hole of the same block. In each freezing period, the temperature within the stone block was decreased continually from 20 to −8 °C for 2 h (Figure 1c). For the next 4 h, the temperature was linearly decreased until −12 °C was reached. The thawing period consisted of water flooding and controlled air-heating segments. The flooding stopped when the water level inside the device was 20 cm above the plastic cup. After that, the water was drained and the controlled heating segment started. The temperature was linearly raised for 5 h and 30 min until 20 °C was reached. The test was over after 30 freeze–thaw cycles. Specimens were visually checked using the scale presented in Table 2. If the noticed damage was larger than damage code “3”, the specimen had failed the test and was characterised as not frost-resistant.

## 3. Results and Discussion

**Figure 2:**
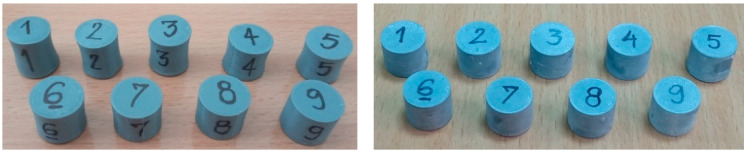
Specimens after annealing in salt (**left**) and alabaster (**right**).

The geometric characteristics of all cylinders before and after annealing in salt and alabaster are shown in Table 3. The presented results are the average values of the three repetitive measurements. The repeatability of the results, according to the calculated standard deviations was very good, i.e., below 0.2%. The specimens’ dimensional characteristics after the liquid nitrogen immersion (LN) test were approximately the same as in the case of as-printed specimens and therefore were not presented and discussed further. The significant shrinkage of cylinder specimens (S1–S6) is registered only for the salt treatment. Visual inspection (Figure 2) has revealed the presence of NaCl grains on the upper and bottom surfaces as well as a continuous white residue of alabaster around the cylinder.

**Figure 3:**
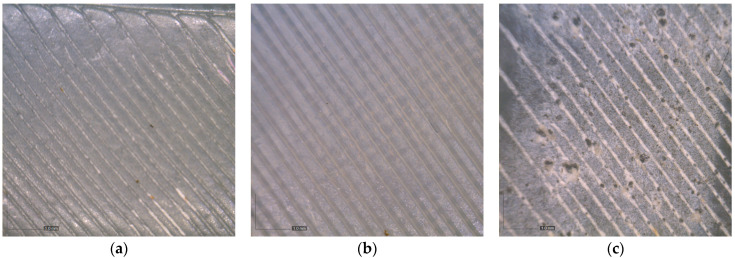
Bottom surface of the specimens: (**a**) NT, (**b**) SA and (**c**) AL; optical images (60×).

This is an expected result and is related to the processing of the salt preparation and the fact that alabaster was not ground. The temperature during annealing in salt was higher, resulting in a higher deformation of the specimens. In addition, during annealing in alabaster, the deposited alabaster acts on the surface of the specimen like an insulation material which prevents its deformation. The deposition of alabaster on the specimen surface (visually registered white residue) was additionally confirmed on optical images. The corresponding bottom surface images related to the S9 NT, SA and AL specimens are given in Figure 3 as an example. It is evident that NaCl was mostly present in the surface raster gaps. In this case, the protected salt layer was not registered on the specimen surface.

The deviation from the starting geometry reveals an interesting pattern which is connected to the raster printing parameter and the annealing exposure time. This effect is mostly visible for the 0.1 mm raster line. In addition, the presence of NaCl grains within the specimen was registered and confirmed on an SEM/EDS microscope, while the presence of alabaster was not noticed.

**Figure 4:**
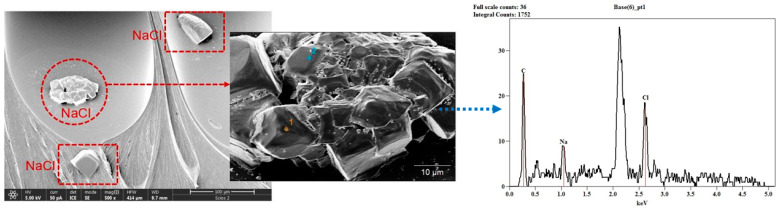
NaCl grains on the surface of S9 specimen (annealed in salt); SEM image (500×) with corresponding EDS.

The corresponding EDS spectrum, as well as the SEM image of the fractured S9 specimen (specimen annealed in salt), is given as an example in Figure 4. The most interesting and unexpected observation was the registration and validation of NaCl grains on and within the printing lines. The same observation was registered on all SA specimens.

**Figure 5:**
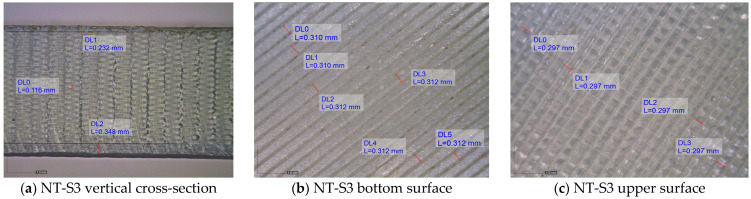

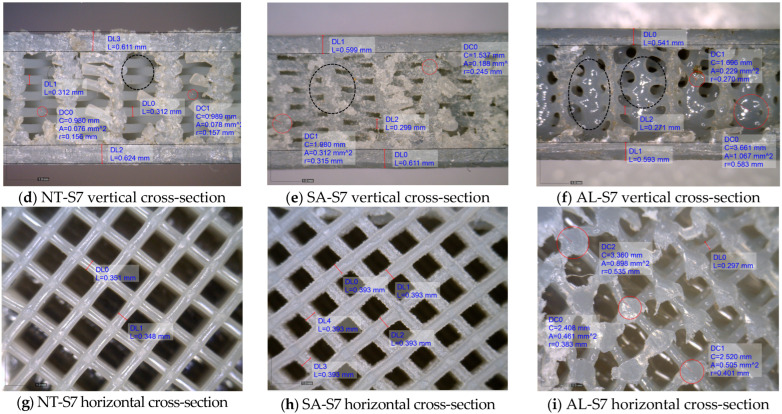
Vertical and horizontal cross-sections of as-printed and thermally treated specimens; SEM images (60×).

The manufacturer of the FDM printer defines that the print temperature is increased by 5 °C only during the formation of the first layer. This increases the stability of the printing structure. In addition, due to the open printer design, the cooling process is not controlled. This means that the temperature and gravitation gradients are established from the top to bottom surface of the specimen. The vertical and horizontal cross-sections of cut rectangular specimens NT-S3, NT-S7, AL-S7 and SA-S7 are presented in Figure 5 as an example.

The simultaneous actions of temperature and gravitation have an additional strengthening effect on the specimens’ bottom surface. The theoretical bottom and upper NT-S3 specimen surfaces consisted of two layers whose thickness was set to 0.1 mm. If the registered values of the bottom and upper surface are compared with the theoretical one, it can be seen that both surfaces were increased (Figure 5a–c). The increase was 74 and 16% for the bottom and the upper surface, respectively. The set printing quality demands define that the deviation between the achieved and theoretical surface dimension is 15%. The difference between the bottom and upper surface is the same as the registered thickness of all other printing layers. This means that the printing quality is uniform through the vertical cross-section of the NT-S3 specimen. This is also confirmation that during the FDM printing process, the bottom surface consists of three layers instead of two. Additionally, this is the explanation of why the default increase in temperature settings is only applied during the first layer formation. This pattern was also valid for each other NT specimen printed with the 0.1 mm raster line.

From Figure 5d–f, it can be seen that the measured thickness for both surfaces is highest in the case of the NT-S7 specimen. The theoretical specimen surface consists of two layers whose thickness is 0.3 mm. The thickness increase in comparison to the theoretical one was 4 and 1.83% for the bottom and upper surface, respectively. The thickness deviation of thermally treated specimens in comparison to the theoretical one was +1.83 (SA) and −1.16 (AL) % and −0.02 (SA) and −9.83 (AL) % for the bottom and upper surface, respectively. The difference in the measured bottom and upper thickness was 0.026, 0.012 and 0.052 mm for the NT, SA and AL specimens, respectively. This is closely related to the applied thermal treatment.

In the case of the NT specimen, each layer height was uniform through the vertical orientation (the black circle in Figure 5d). It means that the diameter of the printing line cross-section was constant. In the case of the specimen thermally treated with salt (NaCl), the vertical cross-section was additionally strengthened (the black circle in Figure 5e). It is also visible that the dimension of the knots cross-section was increased. In the case of NaCl annealing, the strengthening effect was caused by two factors. The first is related to the physical deposition of the salt on the specimen surface, while the second is connected to the NaCl diffusion inside the specimen. Since the exposure time is not so high (30 min) and the temperature is near the melting point, the reason for surface thickness reduction is obvious. In this case, the printing pattern was kept in horizontal and vertical cross-sections (Figure 5e,h). Compared to the untreated specimen, the raster lines uniformly increased in horizontal cross-section by approximately 11%.

**Figure 6:**
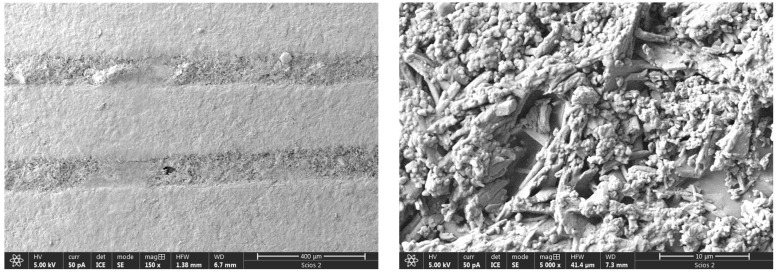
Microstructure of the specimen S7 treated in alabaster (AL); SEM images.

The thermal strengthening effect on the specimen annealed with alabaster was related to the formation on the specimen surface of the protective alabaster layer, as given in Figure 6. Interestingly, the print pattern was kept only in the horizontal cross-section (Figure 5i). Even though the exposure time and the annealing temperature during thermal treatment were severe (3 h), the alabaster protected the specimen from complete melting. Local material softening led to a decrease in horizontal printing lines and diffusion from adjacent lines to the overlapping knots. In the vertical direction, the material was strengthened. This effect is more profound on the bottom surface than on the upper surface (black circles in Figure 5f). Compared to the untreated specimen, the cross-section lines of the AL specimen were reduced by approximately 15%, as shown in Figure 5i. It is evident that the printed layer connection pattern consisted of tick parallel approximately rectangular bars, and that the printing lines between those bars in each layer are thinner than in the case of NT specimens. In addition, the geometric parameters (cylinder height) given in Table 3 are in line with these findings.

**Figure 7:**
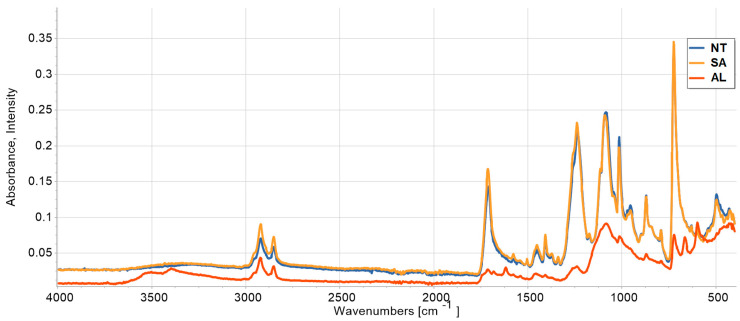
FTIR spectra for NT-S3, SA-S3 and AL-S3 specimens.

**Figure 8:**
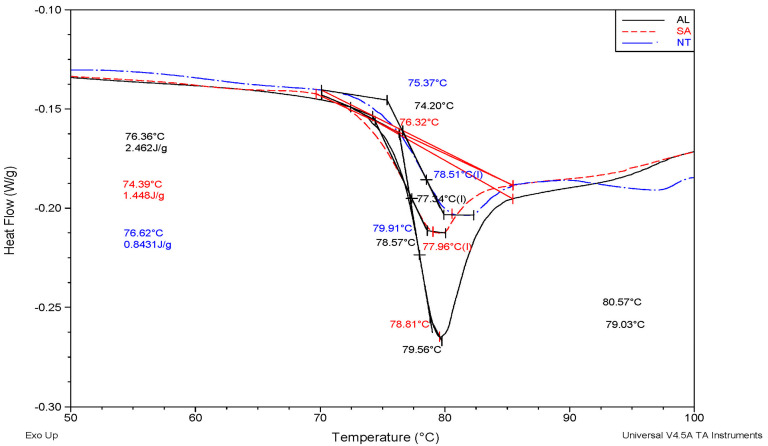
DSC curves for NT-S3, SA-S3 and AL-S3 specimens.

FTIR and DSC results are given in Figure 7 and Figure 8 for NT-S3, SA-S3 and AL-S3 specimens, which were given as an example. FTIR spectra for the SA-S3 specimen has larger intensity in comparison to the NT-S3 specimen at wave lines 2921, 2852, 1712, 1577, 1452 and 1238 cm^−1^. The opposite situation is registered at wave lines 1091, 1015 and 955 cm^−1^. The spectra for the AL-S3 specimen are different to the NT-S3 and SA-S3 specimens at wave lines 3502, 3396, 1618, 1540 and 1682 cm^−1^ (which correspond to the alabaster). The intensity of the whole spectra for the AL specimen is lower than the corresponding spectra for the NT and SA specimens. This is caused by the presence of the protective alabaster layer on this specimen surface. The overall spectra for all specimens suggest that the polymer matrix has not changed. During annealing, salt diffused and also deposited inside the material. The presence of C–H stretched alkanes was registered at 2921 and 2852 cm^−1^. The pick at 1712 cm^−1^ is related to the ketone group, while the aromatic rotations are seen at 1452 cm^−1^. The peaks at 1240, 1084 and 1013 cm^−1^ are caused by the stretching of the C–O group. The C–H bending vibration of benzene derivatives is registered at 871 and 793 cm^−1^. The shape of FTIR curves, as well as the identified peaks registered in Figure 7, are similar to the FTIR spectra for PETG material reported elsewhere [49,50,51].

By analysing the DSC curves, it can be seen that the glass transition temperature (Tg) values of the NT-S3, SA-S3 and AL-S3 specimens are 78.51, 77.96 and 77.34 °C, respectively. The energies required to raise the specimens’ temperature to the Tg value were also calculated. These values were 0.8431, 1.448 and 2.462 J/g for the NT-S3, SA-S3 and AL-S3 specimens, respectively. In comparison to the as-printed condition, the annealing process decreased the Tg and improved the thermal stability by 1.7 and 2.9 times for SA and AL specimens, respectively. These results are in line with the discussed annealing strengthening mechanisms. It is interesting that trend in decreasing Tg, as well as improvement in thermal stability, was also found for materials which were reinforced with graphene [49]. The pro-longed softening process and increased thermal stability were caused by the salt presence above, around, and in the raster lines of the SA specimens. In the case of the NT specimens, the protective alabaster layer was responsible for the increased thermal stability. In both cases, this allowed for a better connection of the print layers in vertical and horizontal directions.

**Figure 9:**
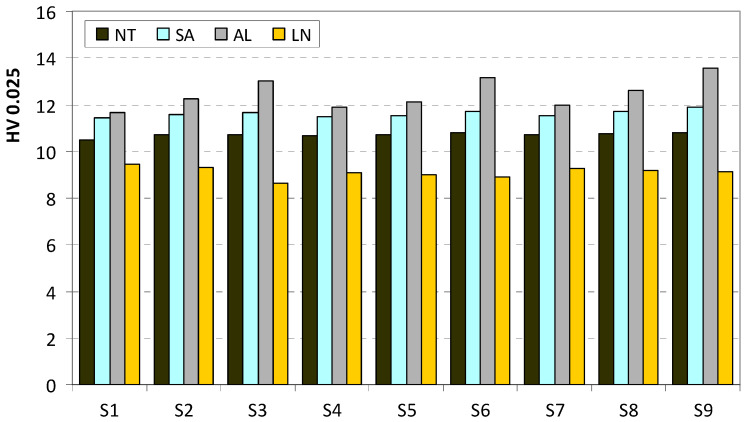
Microhardness values for as-printed (NT), salt-treated (SA), alabaster-treated (AL) and liquid nitrogen-treated (LN) specimens printed with different printing parameters.

The hardness results are presented in Table 4. The presented results are the average values of the five repetitive measurements. The repeatability of the results according to the calculated standard deviations was good, i.e., below 5%. Higher hardness was registered on the bottom surface for most of the specimens, regardless of the applied treatment. This is in accordance with the FDM printing process and the presence of one additional printing layer on the specimens’ bottom surface (Figure 5). The differences between the bottom and upper surface were not that different, and the average values of microhardness are calculated and presented in Figure 9.

From Figure 9, it can be seen that thermal treatments in salt and alabaster increased the hardness values of the as-printed samples. Treatment in salt increased microhardness by approximately 8%, while the alabaster treatment increased it by 16%. On the other hand, liquid nitrogen decreased the average microhardness by approximately 15%. This decrease was in correlation with the long exposure time to the liquid nitrogen and a low degree of PETG crystallinity (approximately 3%) [52].

**Figure 10:**
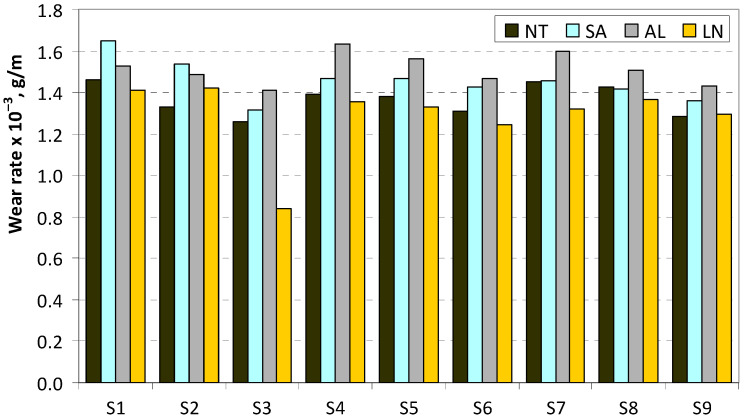
Abrasive wear rate values for as-printed (NT), salt-treated (SA), alabaster-treated (AL) and liquid nitrogen-treated (LN) specimens printed with different printing parameters.

The wear test results are presented in Table 5. The presented results are the average values of the four repetitive measurements. The repeatability of the results, according to the calculated standard deviations, was good, i.e., below 10%. It is evident that there was a very small difference between the bottom and upper surface values, so average values were calculated and are presented graphically in Figure 10. The abrasion wear test was performed for 40 m, which is enough to minimise the effect of the higher hardness of the bottom surface, i.e., the surface layers with higher hardness were worn out at the beginning of the test and did not influence the total wear rate too much. Regarding the printing parameters of layer height and density, only density influenced wear results, i.e., specimens with higher density showed a lower wear rate. Lower density is connected with a lower area of specimen coming into contact with the counterbody (abrasive paper), which induces higher specific pressure and consequently higher wear. A similar connection was noticed by Srinivasan et al. [53], who showed that when the infill density increases, the surface becomes smoother, i.e., the bearing area was bigger. The wear resistance was not in correlation with the hardness values, which is relatively surprising since abrasive wear is usually in direct correlation with hardness. In our case, the specimens with higher hardness had higher wear as well. The salt- and alabaster-treated specimens (SA and AL specimens) showed the highest wear rates, while the specimens treated with liquid nitrogen, although showing the lower hardness, showed the highest wear resistance. This phenomenon needs further investigation.

The results of the hardness and wear testing indicate that the most interesting printing series are S1, S3, S7 and S9. This is the reason that further material characterisation (tensile test and impact test) was did only on specimens with this raster line and infill density combination. The results of the Charpy impact test are given in Table 6. The presented results are the average values of the four repetitive measurements. The repeatability of the results, according to the calculated standard deviations, was good, i.e., below 10%. The lowest Ut value was registered for the LN specimens. The relative decreases in the Ut value for LN-S1 and LN-S3 compared to the NT were 12 and 31%, respectively. In the case of the LN-S7 and LN-S9 specimens, the Ut decreases were 29 and 41%, respectively. In the case of extreme temperature exposure, the lower value of the raster line provides higher impact resistance for specimens with the same infill density. On the other hand, salt and alabaster treatments have a positive effect on impact resistance. The highest values are registered for AL specimens. The protective alabaster layer is more uniform on specimens printed with the 0.3 mm raster line. For example, the Ut value for the AL-S9 specimen is approximately three times higher than that of the NT-SA9 specimen. The relative increase in Ut values for specimens SA-S1 and SA-S3, in comparison to the NT specimen, was approximately 19%. In the case of the SA-S7 and SA-S9 specimens, the relative increase in Ut was around 4%. This is a clear indication that for specimens printed with a large infill density, the impact resistance is not drastically improved with raster line increase. If specimens are printed with lower infill density, a strong correlation between raster line increase and impact resistance is observed.

**Figure 11:**
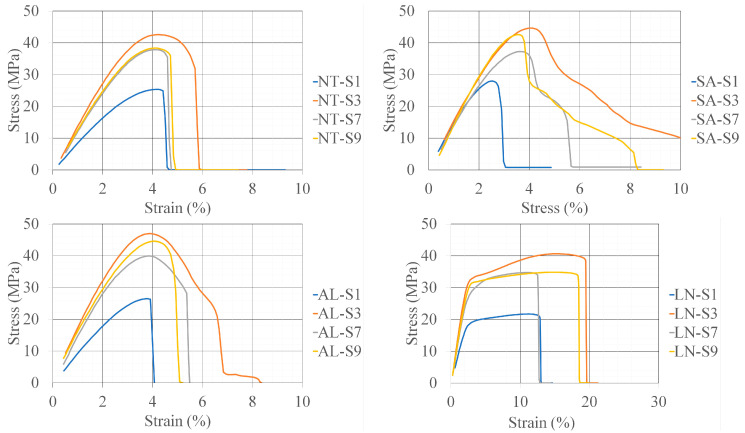
Tensile test results.

The results of the tensile test are given in Table 7 and Figure 11 (stress–strain diagrams). For the LN specimens, the maximum stress was decreased compared to the corresponding NT specimens. A maximum decrease of 14.29% was registered for the S1 specimen. From Table 7 and Figure 11, it is obvious that LN specimens can withstand stresses for a longer period, in the range of 80–95% of the maximal registered stress value, than the corresponding NT, SA or AL specimens. Even though the maximal stress decrease in comparison to that in NT is evident, the ability of a material to withstand “useful” stresses is drastically improved, and this is the reason that the extreme temperature-exposing process can be regarded as a strengthening treatment.

**Figure 12:**
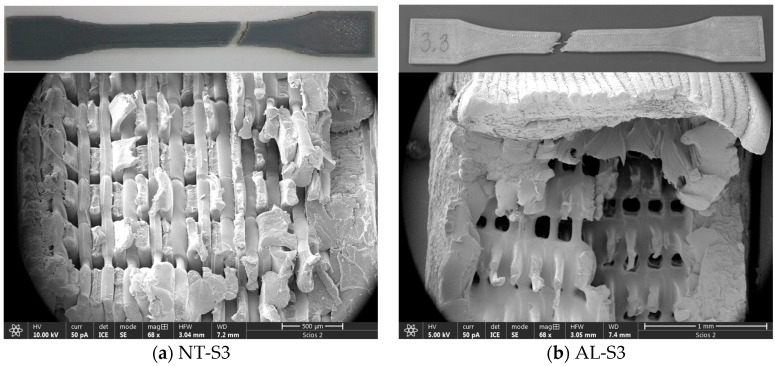
Image of the fractured specimens. SEM images (bottom) are cross-section details of the specimens after the tensile test.

Thermal treatments also have a positive effect on the mechanical properties. If maximal stress is used as a comparison criterion, an interesting pattern, valid in each treatment, was revealed. The best-rated specimens had a raster line of 0.1 mm and an infill density of 90%, while the worst-rated specimens were printed with a raster line of 0.3 mm and an infill density of 30%. The macroscopic view of the specimens NT-S3 and AL-S3 after the tensile test and the cross-section of NT-S3 and AL-S3 specimens are given in Figure 12 as an example. Theoretically, specimens printed with a raster line of 0.1 mm will have three times more lines than in the case of specimens printed with a raster line of 0.3 mm due to the fact that the test specimens had the same geometry. Because a force was applied in the direction of the printing lines, maximal stress values were expected in the case of specimens printed with more lines and a higher infill density. In addition, the strain values of specimens printed with the same raster line should be approximately the same in the case of NT specimens. Interestingly, the shape of the NT stress–strain curves deviated in the case of the SA and AL specimens, except for one printed with a raster line of 0.1 mm and an infill density of 30%. The registered deviations are closely connected with the explained strengthening mechanisms (Figs. 5f and 12b). The shape of the AL-S3, SA-S3, SA-S7 and SA-S9 specimens’ stress–strain curve is different in comparison with the corresponding NT specimen curves. In these cases, the material properties gradually changed from brittle to ductile.

All specimens subjected to the long-term durability frost resistance test were classified as frost resistant after a visual check. Interestingly, the damaged code “0” was assigned to all thermally treated specimens, which was not the case with the as-printed specimens. On the as-printed specimens, one or more hairline cracks were formed on the surface, corresponding to the damage code “2”. This is in correlation with results for PETG specimens presented by Sedlak et al. [14].

After comprehensive testing of the identified important characteristics and analysis of the results, it was concluded that PETG specimens annealed in salt (SA-S3) and alabaster (AL-S3) showed the best overall properties. Since the results for these two samples were very similar, further comparison and rating were on the thermal treatment energy requests (lower exposure times and reduced energy consumption), and specimen SA-S3 was chosen for comparison with the ABS samples. The measured important characteristics of standard ABS material and the specimen SA-S3 made of alternative (PETG) material for drone spare part manufacturing are summarised in Table 8.

To neglect the influence of layer height and infill density, a comparison between ABS-NT-S3 and PETG-SA-S3 was made. The PETG material was better in all tested properties, i.e., increase in hardness by 2.6%, decrease in wear rate by 45.4% and increase in Charpy impact toughness by 33.7%. Maximum tensile stress was 49.6% higher and the modulus of elasticity was reduced by 8.5%, which is favourable in applications where flexibility is required.

**Figure 13:**
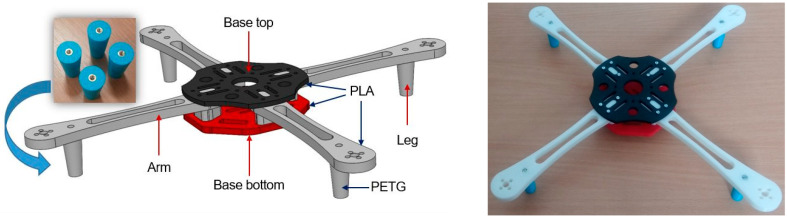
FDM printed drone prototype: 3D model (**left**) and manufactured model (**right**).

As a final step, a prototype drone frame was printed (Figure 13), with legs made of PETG material printed and treated in the same way as the specimen SA-S3. The performance of this prototype, i.e., testing of the material in real environment conditions, was not performed in this study. It is just an illustration that a drone frame could be produced as a modular construction with legs made of PETG material as an alternative to ABS material. A drone’s arms and legs are usually considered as one compact spare part. In this study, the arms and legs were designed to be two unique spare parts. Using this concept, frame modularity was increased and serviceability was enhanced. This is particularly important since the global demands for drones are exponentially growing. According to Maricic et al. [54], drone production is projected to grow by 11% annually.

## 4. Conclusions

This paper investigated PETG as an alternative material in the manufacture of drone spare parts. Hardness, abrasive wear, impact resistance, tensile strength and durability were identified as important characteristics in the testing of PETG materials. All characteristics were determined experimentally on as-printed, annealed in salt and alabaster, and liquid nitrogen-immersed specimens. In this regard, thermal treatments have been shown to positively influence all the characteristics of printed spare parts. The annealing in salt was applied to the printed legs. The results of the specimens annealed in salt and the ones annealed in alabaster were similar, so the economic reasons were decisive. From an economic point of view, annealing in salt is better due to the shorter process time and lower energy consumption. In addition, salt can be used multiple times, unlike alabaster, which becomes municipal waste after treatment. In comparison with ABS material, PETG material annealed in salt showed improvement in all tested properties, indicating that PETG could be an alternative to ABS material for the production of drone spare parts.

In future research, a comparison of as-printed and reinforced PETG should be made. If the salt treatment were to be repeated, it would be desirable to reduce the exposure time from 30 to 15 min. Another long-term durability test which includes weathering, UV radiation, humidity and temperature agents should be applied. In addition, thorough tests should be conducted based on the designed drone frame. Theoretical FEA analysis with physical testing should show drone frame static load limits, i.e., whether it can ensure reliability in complex operating conditions.

## Figures and Tables

**Table 1:** Printer set values.

Fixed Parameter	Value								
Top and bottom patterns	4								
Top/bottom layers	2								
Infill pattern	zigzag								
Raster angle (infill line direction)	+45°/−45°								
Printing temperature	230 °C								
Plate temperature	85 °C								
Print speed	50 mm/s								
Build plate adhesion type	skirt								
Variable parameter	Series								
	S1	S2	S3	S4	S5	S6	S7	S8	S9
Raster line (layer height) (mm)	0.1	0.1	0.1	0.2	0.2	0.2	0.3	0.3	0.3
Infill density (%)	30	60	90	30	60	90	30	60	90

**Table 2:** Visual frost scale.

Damage Code	Damage Description
0	unchanged sample
1	very small damage which does not affect the sample
2	one or more hairline cracks (≤0.1 mm) or delamination (≤3 mm^2^)
3	one or more hairline cracks (≤0.1 mm) or delamination (≤1 mm^2^)
4	sample is split into two parts
5	sample is completely destroyed

**Table 3:** Dimensional characteristics with corresponding standard deviations (lower value).

**Cylinder Diameter (mm)**	**S1**	**S2**	**S3**	**S4**	**S5**	**S6**	**S7**	**S8**	**S9**
NT	16.08	16.05	16.00	16.02	16.03	16.03	16.01	16.02	16.03
	0.01	0.01	0.01	0.01	0.01	0.01	0.00	0.01	0.01
SA	15.25	15.15	15.35	15.05	14.99	14.8	14.75	15.06	15.26
	0.01	0.01	0.01	0.01	0.01	0.01	0.01	0.01	0.01
AL	15.83	15.85	15.96	15.84	15.87	15.92	15.82	15.88	15.95
	0.01	0.01	0.01	0.01	0.01	0.01	0.01	0.01	0.01
**Cylinder Height (mm)**	**S1**	**S2**	**S3**	**S4**	**S5**	**S6**	**S7**	**S8**	**S9**
NT	12.98	12.98	13.09	12.95	12.93	12.95	12.89	12.92	12.96
	0.01	0.01	0.02	0.01	0.01	0.02	0.01	0.01	0.00
SA	13.15	12.71	12.96	13.21	13.26	13.3	13.27	13.26	13.25
	0.01	0.01	0.01	0.01	0.01	0.01	0.01	0.01	0.01
AL	12.72	12.71	12.96	12.8	12.69	12.75	12.69	12.75	12.92
	0.01	0.02	0.02	0.02	0.01	0.02	0.03	0.00	0.01

NT—as-printed material (without treatment); SA—salt thermal treatment; AL—alabaster thermal treatment.

**Table 4:** Specimen microhardness (HV 0.025) with corresponding standard deviation (lower value).

Specimen	Bottom Surface				Upper Surface			
	NT	SA	AL	LN	NT	SA	AL	LN
S1	10.55	11.53	11.65	9.60	10.41	11.32	11.65	9.31
	0.18	0.32	0.32	0.28	0.22	0.18	0.25	0.18
S2	10.68	11.57	12.07	9.35	10.73	11.56	12.40	9.28
	0.25	0.38	0.38	0.17	0.25	0.27	0.35	0.19
S3	10.76	11.72	13.00	8.85	10.67	11.57	13.00	8.40
	0.29	0.26	0.33	0.26	0.31	0.18	0.40	0.22
S4	10.54	11.43	11.70	9.04	10.79	11.55	12.10	9.14
	0.22	0.36	0.38	0.25	0.25	0.27	0.30	0.27
S5	10.64	11.59	12.06	9.02	10.78	11.50	12.16	8.98
	0.18	0.39	0.36	0.10	0.32	0.26	0.23	0.29
S6	10.82	11.73	13.13	9.08	10.82	11.65	13.13	8.74
	0.35	0.26	0.39	0.25	0.31	0.20	0.12	0.23
S7	10.55	11.46	11.77	9.39	10.88	11.62	12.14	9.14
	0.35	0.33	0.27	0.24	0.33	0.20	0.18	0.19
S8	10.89	11.73	12.63	9.38	10.61	11.71	12.63	8.98
	0.27	0.36	0.23	0.19	0.28	0.21	0.39	0.05
S9	10.92	11.89	13.55	9.34	10.70	11.90	13.57	8.95
	0.31	0.25	0.25	0.11	0.25	0.30	0.35	0.10

NT—as-printed material (without treatment); SA—salt thermal treatment; AL—alabaster thermal treatment; LN—liquid nitrogen treatment.

**Table 5:** Specimen wear rate results × 10^−3^ (g/m) with corresponding standard deviation × 10^−3^ (g/m) (lower value).

Specimen	Bottom Surface				Upper Surface			
	NT	SA	AL	LN	NT	SA	AL	LN
S1	1.47	1.61	1.52	1.41	1.44	1.68	1.53	1.41
	0.03	0.04	0.06	0.14	0.14	0.03	0.12	0.11
S2	1.35	1.52	1.48	1.44	1.31	1.55	1.49	1.40
	0.07	0.06	0.11	0.03	0.09	0.12	0.11	0.04
S3	1.26	1.31	1.41	0.86	1.25	1.31	1.42	0.82
	0.07	0.08	0.04	0.06	0.06	0.10	0.03	0.06
S4	1.38	1.42	1.66	1.34	1.40	1.52	1.60	1.36
	0.04	0.03	0.11	0.11	0.10	0.08	0.09	0.05
S5	1.39	1.45	1.60	1.31	1.37	1.48	1.53	1.35
	0.08	0.04	0.11	0.10	0.03	0.07	0.07	0.07
S6	1.31	1.42	1.45	1.24	1.31	1.44	1.48	1.25
	0.06	0.05	0.11	0.09	0.07	0.09	0.06	0.06
S7	1.45	1.45	1.59	1.32	1.46	1.47	1.60	1.33
	0.05	0.10	0.10	0.06	0.08	0.09	0.07	0.04
S8	1.44	1.40	1.50	1.36	1.41	1.44	1.51	1.37
	0.07	0.08	0.12	0.13	0.08	0.04	0.08	0.11
S9	1.29	1.35	1.44	1.29	1.28	1.37	1.42	1.30
	0.06	0.13	0.12	0.08	0.11	0.11	0.07	0.08

NT—as-printed material (without treatment); SA—salt thermal treatment; AL—alabaster thermal treatment; LN—liquid nitrogen treatment.

**Table 6:** Charpy impact test results with corresponding standard deviations (lower value).

Specimen	Charpy Impact Toughness, Ut (kJ/m^2^)			
	NT	SA	AL	LN
S1	14.61	17.30	20.20	12.76
	0.10	1.32	1.59	0.46
S3	27.39	32.59	39.61	18.82
	0.07	3.06	2.67	0.55
S7	8.26	8.64	20.87	5.80
	0.08	0.14	0.49	0.22
S9	39.71	41.03	130.8	23.52
	2.18	0.28	2.20	2.08

NT—as-printed material (without treatment); SA—salt thermal treatment; AL—alabaster thermal treatment; LN—liquid nitrogen treatment.

**Table 7:** Tensile test results.

Treatment	Specimen	*F*_max_ (N)	Max. Stress, *σ* (MPa)	Strain at Max. Stress, *ε* (%)	Modulus, E (MPa)
NT	S1	801	25.33	4.22	8.20
	S3	1346.5	42.58	4.23	13.43
	S7	1197	37.85	4.12	12.12
	S9	1212.5	38.34	4.15	12.21
SA	S1	1175	27.96	3.23	13.25
	S3	1688.5	44.66	4.01	15.10
	S7	1409.5	37.28	3.84	13.68
	S9	1363.5	48.87	4.01	15.45
AL	S1	837.5	26.48	3.79	9.44
	S3	1485.5	46.98	3.91	15.95
	S7	1263	39.94	3.89	14.25
	S9	1410	44.01	4.05	14.57
LN	S1	686.5	21.71	11.34	7.13
	S3	1285.5	40.65	15.28	14.21
	S7	1099	34.74	11.43	11.66
	S9	1001.5	34.84	14.72	12.74

NT—as-printed material (without treatment); SA—salt thermal treatment; AL—alabaster thermal treatment; LN—liquid nitrogen treatment.

**Table 8:** Comparison of the ABS and PETG material.

Specimen	HV 0.025	Wear Rate × 10^−3^ (g/m)	Ut (kJ/m^2^)	*F*_max_ (N)	Max. Stress, *σ* (MPa)	Strain at Max. Stress, *ε* (%)	Modulus, E (MPa)
ABS-NT-S1	10.65	2.68	9.23	517	16.26	2.46	8.96
ABS-NT-S3	11.42	2.40	24.38	950	29.86	2.35	16.51
ABS-NT-S7	10.47	3.01	12.70	849.5	31.19	2.45	17.09
ABS-NT-S9	11.62	3.60	18.80	992.5	29.77	2.26	16.89
PETG-SA-S3	11.72	1.31	32.59	1688.5	44.66	4.01	15.10

NT—as-printed material (without treatment); SA—salt thermal treatment.

## Data Availability

The original contributions presented in the study are included in the article, and further inquiries can be directed to the corresponding author.
